# Correlative Imaging and super resolution microscopy studies reveal complexities in determining live-dead state of bacteria

**DOI:** 10.1016/j.bioflm.2025.100302

**Published:** 2025-07-03

**Authors:** Jiaqi Luo, Rasmita Raval

**Affiliations:** a The Open Innovation Hub for Antimicrobial Surfaces, Surface Science Research Centre, Department of Chemistry, University of Liverpool, Liverpool, L69 3BX, UK; b National Biofilms Innovation Centre, UK

**Keywords:** Correlative light electron microscopy, Structural illumination microscopy, SYTO 9, Propidium iodine, Live-dead, Staphylococcus aureus

## Abstract

Imaging techniques are widely used to determine the physiological state of bacterial cells and provide an important platform for antibacterial evaluation in biofilms research. The commercial counter-staining SYTO 9 – propidium iodine kit is a popular choice for viability studies, with cell membrane damage due to antimicrobial action leading to replacement of the SYTO 9 dye with propidium iodine. This study investigates the live-dead state of cells in early-stage *Staphylococcus aureus* biofilms using correlative Fluorescence Microscopy (FM), Scanning Electron Microscopy (SEM) and super resolution Structural Illumination Microscopy (SIM). Correlative imaging data obtained at the single-cell level show that the physiological states of individual bacterial cells indicated by the SYTO 9 – propidium iodine counterstain in FM does not correlate directly with the detailed cell morphology observed by SEM. In addition, SIM was used to map sub-cellular distributions of SYTO 9 – propidium iodine dyes within single cells and revealed greater complexity than hitherto assumed, with 4 different cell-states identified, including double-stained ones and those where SYTO-9 is bound to substances at the cell perimeter. With this knowledge, we present ternary plots to illustrate the significant impacts of this complex staining behaviour on underestimation of cell-membrane damage due to antimicrobial actions and, thus, overestimation of bacterial survival rate in biofilms research.

## 1 Introduction

Biofilms represent the dominant mode of life for bacteria, forming communities of microorganisms held within a matrix and commonly adhered to a surface [1]. The global economic impact of biofilms has been estimated to be in excess of $5000 bn a year [2]. In health, they are implicated in over 80 % of chronic microbial infections in the human body [2] and play an important role in driving antimicrobial resistance (AMR) [3]. Considerable research and innovation efforts are, therefore, directed towards creating anti-infective and anti-biofilm technologies to combat the deleterious effects of biofilms that colonise surfaces across multiple sectors and applications, e.g. medical devices such as catheters, implants and endotracheal tubes, oral care products and disinfection in healthcare, to food safety and marine biofouling [2]. In order to progress these ambitions, it is important to detect and assess bacterial biofilms, for instance, to visualise them and to understand the live-dead states of the cells via imaging techniques. Two major techniques in the field are scanning electron microscopy (SEM) and fluorescence microscopy (FM). The SEM technique images both the bacterial cells and the surfaces they are attached to. In medium magnification (field of view about 50 μm), the distribution of bacterial cells can be studied, as well as other large-scale features or events that occur on the surface. At higher magnifications, SEM can reveal detailed features at a sub-micron or nano scale, and thus has the capability to examine subtle changes. Consequently, SEM is widely used for observation of individual cells [[4], [5], [6], [7]], where the morphology of cells and cell membrane integrity are regarded as key indicators of their state. For example, it is common to assign undeformed and intact cells to a healthy state and classify deformed or membrane-damaged cells as compromised cells [5,6].

Bacterial cells in biofilms can also be imaged by combined use of fluorescent dyes and fluorescence microscopy (FM). A widely-used approach is using a LIVE/DEAD Viability kit that is composed of a pair of dyes, SYTO 9 and propidium iodine (PI), which bind to nucleic acids [[8], [9], [10], [11]]. SYTO 9 is able to permeate the cell membrane and, therefore, can stain the nucleic acids within the bacterial cells and emits fluorescent green. PI is only able to permeate a compromised membrane, however, once it does, the nucleic acids will be labelled by PI due to its higher affinity compared to SYTO 9 and the cell emits fluorescent red. Besides, as the emission spectrum of SYTO 9 overlaps with the excitation of PI, fluorescence resonance energy transfer (FRET) can also happen, which quenches the emission of residual SYTO 9 on the PI-labelled nucleic acids. This counterstaining method is used to indicate the membrane integrity of the bacterial cells, which are then classified as either live or dead. However, in practice, FM images do not always show a straightforward binary classification. For example, a phenomenon called double-staining was observed in SYTO 9 – PI studies [8,[12], [13], [14], [15]], where the bacterial cell is simultaneously stained by both dyes. This phenomenon is generally explained either as the incomplete replacement of SYTO 9 by PI in a competitive process [8,15], or arising from the presence of extracellular nucleic acids (eNA) that are stained by PI, while the intracellular nucleic acids remain stained by SYTO 9 if the membrane is intact [14,16,17]. Such observations indicate that the SYTO 9 – PI staining method, which is routinely used to evaluate survival rate or antibacterial efficiency, could over/under-estimate live cells causing false-negative/positive results.

In this paper, we investigate some limitations and complexities that can arise for both techniques. *Staphylococcus aureus* (*S. aureus*) was chosen as a model microorganism, and 24 h lab-grown biofilms on surfaces examined. In the first part of this study, correlative SEM-FM experiments were undertaken to explore the information obtained on the cell state via the two techniques. In the second part, the super-resolution technique of structural illumination microscopy (SIM), which goes beyond the diffraction limit, is utilised to further probe the distribution of dyes within single cells. This work offers new insights into single-stained and double-stained cells and the behaviour of SYTO 9, which could have direct impact on viability assessments using the SYTO 9 – PI co-staining method.

## 2 Results

### 2.1 WFM-SEM correlative studies on SYTO 9-PI co-stained *S. aureus* biofilm

At a starting point for the correlative studies, it is important to understand what information is available from each imaging technique. A conventional widefield fluorescence microscope (WFM) with a numerical aperture of 0.75 and above is able to provide sub-micron lateral resolution and thus distinguish individual bacterial cells which are typically 0.5–5 μm in length [18]. In WFM, bacterial cells can be examined from three main perspectives: the type of dye that stains the bacterial cell, the intensity of the fluorescent emission, and the area the emission comes from. The first can be the easiest one to judge, provided that the crosstalk of dyes (overlap of excitation and emission spectra) is solved by optimising the combination of excitation wavelengths and filter sets. The second and the third aspects require careful experimental consideration since the intensity shown on the final displayed image can be adjusted during post-processing, which might change the size of the fluorescent area.

In contrast to WFM, SEM images can appear to be more straightforward to analyse, as with correct electron beam alignment and detector settings, the morphology and any details around the bacterial cells can be recorded and easily examined. For SEM analysis, three main aspects of interest are size, shape, and membrane integrity of the cells, which can all be imaged at much higher resolution compared to WFM images. Generally, undamaged and undeformed cells are interpreted as intact cells and, by inference, often considered as presenting live or viable cells.

Correlative WFM-SEM approaches have been developed to better compare the information from the two techniques [19]. For instance, correlative studies have been used to investigate the interaction of pathogens and cells [[20], [21], [22]] and to identify fluorescent labelled magnetotactic bacteria [[23], [24], [25]]. Here we report correlative studies at the single bacterial cell level on biofilms grown on a surface using SEM alongside WFM with SYTO 9 – PI co-staining, an approach that has not been previously reported to our best knowledge.

A major consideration of this paper was to investigate bacterial cells that are “single-stained” or “double-stained”. The term “single-stained” refers to cells that show fluorescence mainly in one excitation light and filter set, thus considered as being single-stained by either SYTO 9 or PI. On the other hand, “double-stained” signifies that the fluorescence is detected in both settings, indicating the cells are double-stained by both dyes.

#### 2.1.1 Single-stained *S. aureus* cells

*S. aureus* biofilms were grown on Si wafers for 24 h and co-stained by both SYTO 9 and PI, as described in the experimental section. WFM images were then obtained by 469 nm excitation and by 555 nm excitation, as well as the merged image of both.

**Fig. 1:**
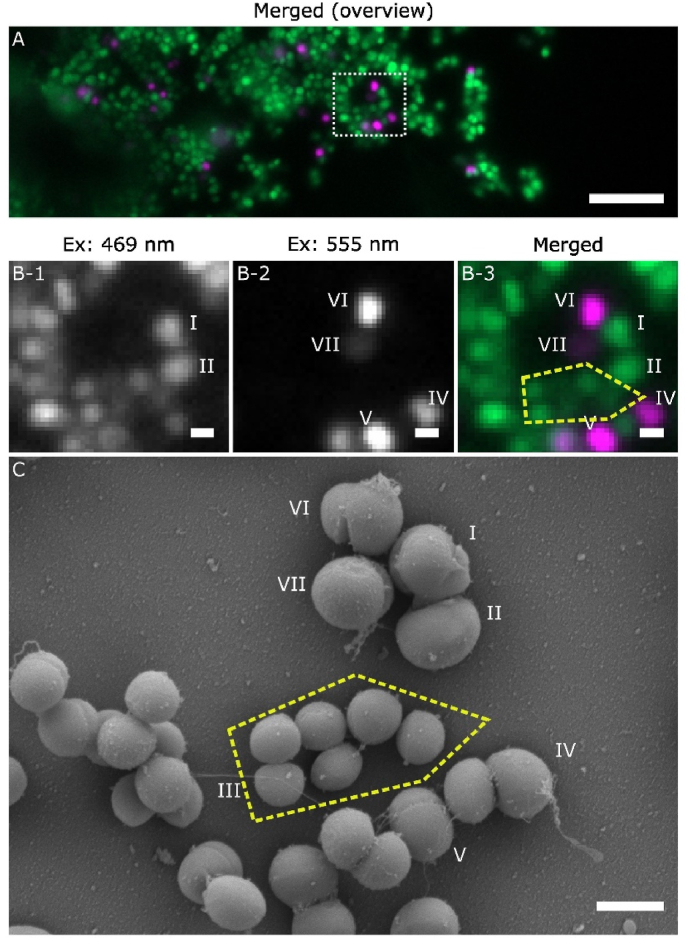
Correlative WFM-SEM data for SYTO 9 – PI stained *S. aureus* biofilms showing single-stained cells. Image (A) is the pseudo-coloured WFM merged image of the emission in the 469 nm excitation (green) and 555 nm excitation (magenta), with the scale bar corresponding to 10 μm. The white rectangle in image (A) indicates the position of the following magnified WFM and SEM images: (B-1) for the emission in 469 nm excitation, (B-2) for the emission in 555 nm excitation, (B-3) is the merged of the above, (C) the high resolution SEM image. Scale bars in images (B, C) correspond to 1 μm. The yellow dotted lines indicate the cells that are used to guide the correlative location between the WFM and SEM images.(For interpretation of the references to colour in this figure legend, the reader is referred to the Web version of this article.)

Fig. 1 A shows the merged image of a large area of *S. aureus* biofilm at the Si surface, showing that the majority of cells were strongly excited by 469 nm but not by 555 nm, suggesting that they were single-stained by SYTO 9, an indicator of intact membrane integrity. In addition, there are a few cells found fluorescent when excited by 555 nm but not by 469 nm, indicating that they were single-stained by PI, a sign of degraded membrane integrity. The preponderance of SYTO 9 single-stained cells is not surprising because the Si wafer does not have an intrinsic antibacterial/antibiofilm effect and the environment provided is optimised for biofilm growth.

A more detailed correlative WFM-SEM analysis was then conducted on defined groups of cells within the white rectangle in Fig. 1 A. Fig. 1 B displays the single channel and merged images of these cells. The same group of cells were later identified and imaged in SEM (Fig. 1 C). It was possible to track individual cells from the WFM to SEM images (e.g. cell labelled I – VII and those within the yellow dotted area), despite some relocation and removal events that can occur during the processing steps when transferring the system between the two characterisation techniques.

This correlative dataset provides interesting insights. First, WFM and SEM images of individual SYTO 9 single-stained cells indicate that cells can inherently be significantly different in size, e.g. comparing cells I and II with those grouped within the yellow dotted lines in Fig. 1 B and C. In addition, the correlated data reveal that the fluorescence intensity of a cell is not related to its size, as demonstrated by cells within the yellow dotted area, which are all of similar size but displaying different intensities. Furthermore, SEM images in Fig. 1 C show that the shapes of the cell can also vary. Alongside the spherical shape, some bacterial cells were found with irregular edges (cell I) or are slightly deformed (cell II).

Correlative SEM data were also obtained for the cells that are single-stained by PI (cells IV - VII), indicating a loss of membrane integrity. However, our correlative data show that they often share similar size, shape, or surface details as those of SYTO 9 single-stained cells around them, e.g. cell IV compared to cell II, cell VI compared to cell I. As observed for the SYTO 9 single-stained cells, the PI single-stained cells can also vary in fluorescence intensity, e.g. cell VI and cell VII show very different fluorescent intensity in WFM, while both appear similar in size and experiencing division in the SEM image. Finer surfaces details are also sometimes observed on these cells: while some are smooth (cell II and most cells in the yellow dotted area), others are observed with unidentified extracellular material around (cells III, IV, VI, and VII).

Overall, our correlative WFM and SEM data show that there is often little direct correlation between the features characterised by WFM and SEM. For example, cells that are stained by different dyes or vary in fluorescence intensity in the WFM images, can look similar and intact in the SEM images, suggesting that the details of a cell shown in an SEM image do not provide a straightforward correlation with the type of dye staining, i.e. the physiological state of the cells.

#### 2.1.2 Double-stained *S. aureus* cells

**Fig. 2:**
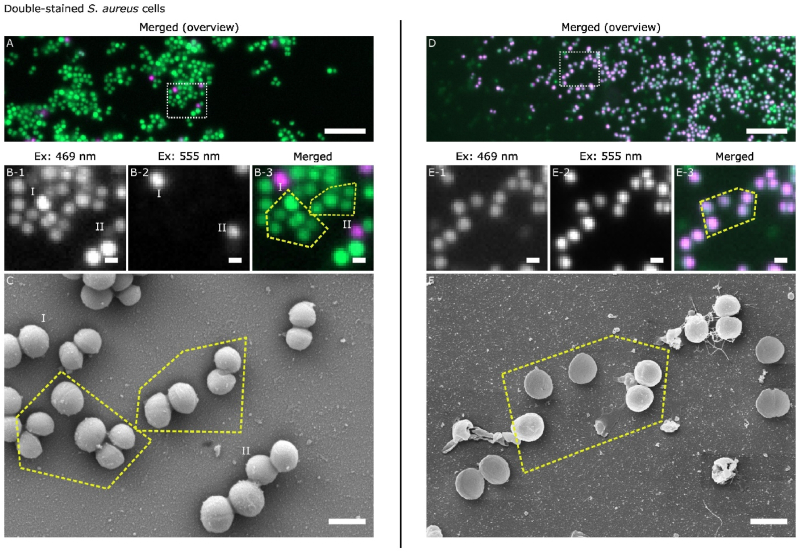
Correlative WFM-SEM data for SYTO 9 – PI stained *S. aureus* biofilms showing double-stained cells. Image (A) is the pseudo-coloured WFM merged image of the untreated *S. aureus* biofilm grown on Si wafer of the emission in the 469 nm excitation (green) and 555 nm excitation (magenta), with the scale bar corresponding to 10 μm. The white rectangle in image (A) indicates the location of the following magnified WFM and SEM images: (B-1) for the emission in 469 nm excitation, (B-2) for the emission in 555 nm excitation, (B-3) is the merged of the above, (C) the high resolution SEM image. Scale bars in images (B-C) correspond to 1 μm. Image (D) is the pseudo-coloured WFM merged image of the 70 % EtOH treated *S. aureus* biofilm grown on coverglass of the emission in the 469 nm excitation (green) and 555 nm excitation (magenta), with the scale bar corresponding to 10 μm. The white rectangle in image (D) indicates the location of the following magnified WFM and SEM images: (E-1) for the emission in 469 nm excitation, (E-2) for the emission in 555 nm excitation, (E-3) is the merged of the above, (F) the high resolution SEM image. Scale bars in images (E–F) correspond to 1 μm. The yellow dotted lines group the cells that are used to guide the correlative relocation between the WFM and SEM images.(For interpretation of the references to colour in this figure legend, the reader is referred to the Web version of this article.)

In addition to the single-stained cells discussed above, there are also cases where cells were found to be fluorescent in both excitation wavelengths displaying a double-stained state. Fig. 2 A shows a merged WFM image of another large area of the *S. aureus* biofilm on a Si surface. Again, most of the cells were single-stained by SYTO 9. However, a closer examination of the cells within the white rectangle in Fig. 2 A and Fig. 2 B reveals that cells I and II, which are strongly excited by 555 nm, are also fluorescent when excited by 469 nm. This suggests that these two cells were double-stained by SYTO 9 and PI. For correlative comparison, SEM images were obtained on the same site as shown in Fig. 2 C. Using the adjacent SYTO 9 single-stained cells highlighted by the yellow dotted lines as guides, it is possible to locate the double-stained cells I and II. In the SEM image, these two double-stained cells look similar and intact compared to the SYTO 9 single-stained cells around them, and no additional features can be identified to distinguish them from others.

In order to obtain more PI-stained cells for further observation, *S. aureus* biofilms were grown on coverglass, and then immersed in 70 % EtOH prior to staining. Ethanol is known to exhibit a significant killing effect on bacteria, producing cells with compromised membranes. Fig. 2 D–F shows the results of correlative WFM-SEM studies undertaken on the co-stained 70 % EtOH treated *S. aureus* biofilms. The overview WFM image in Fig. 2 D demonstrates that, as expected, the biofilm consists of a greater number of PI-stained *S. aureus* cells. The cells within the white rectangle area in Fig. 2 D were then selected for a closer analysis. From the single channel images shown in Fig. 2 E, it is found that many of these PI-stained cells, e.g. those contained within the yellow dotted lines, appeared to be SYTO 9 – PI double-stained, with fluorescent emission for both excitation channels. These cells can also be identified in the correlative SEM image shown in Fig. 2 F, and do not show any unique features associated with the double-stained state. Specifically, they do not display any disrupted morphologies and share similar sizes and shapes as the SYTO 9 single-stained cells discussed in the previous section.

### 2.2 SIM study on double-stained *S. aureus* biofilm

In order to better understand the SYTO 9 – PI double staining phenomenon, structured illumination microscopy (SIM) was utilised. This is a super-resolution microscopy technique, enabling imaging beyond the diffraction limit. This technique uses a patterned light source to illuminate the samples. The same area is illuminated for a number of times, with the pattern changing slightly each time. These raw images are then further reconstructed based on the selected algorithm provided by the instrument manufacturer, resulting in an image with much improved resolution compared to those from WFM or confocal laser scanning microscopy (CLSM).

#### 2.2.1 SYTO 9 – PI co-staining

**Fig. 3:**
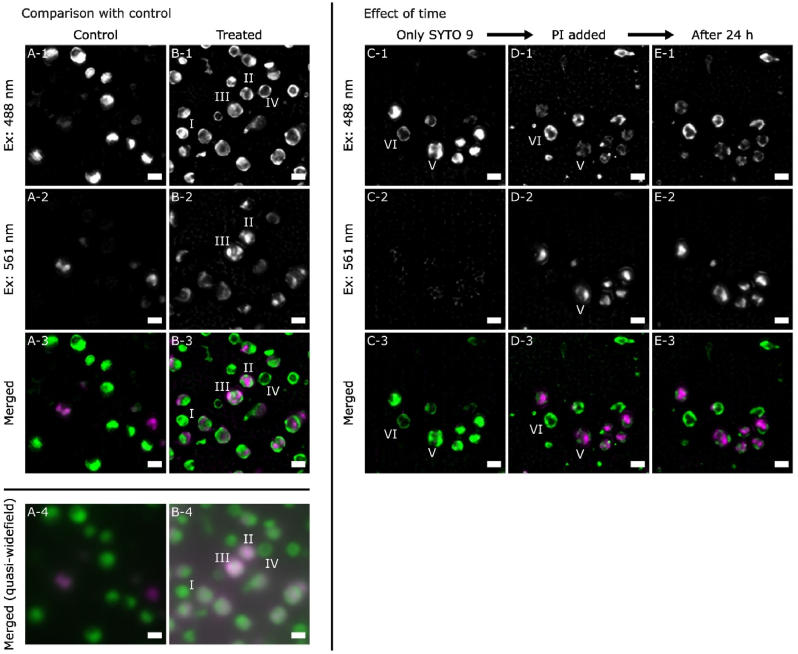
SIM data for SYTO 9 – PI stained *S. aureus* biofilms showing double-stained cells. Left-hand Figure (A & B): Column A shows the representative pseudo-coloured SIM images of a group of SYTO 9 – PI stained untreated *S. aureus* biofilms grown on glass bottom petri dishes, and column B shows the images from the 70 % EtOH treated biofilms. Scale bars in all images correspond to 1 μm. Images (A-1 & B-1) are the emission in 488 nm excitation, images (A-2 & B-2) are the emission in 561 nm excitation, and images (A-3 & B-3) are the merged images of the above two individual excitation (green for 488 nm, and magenta for 561 nm). Images (A-4 & B-4) are the quasi-widefield images generated by the raw images. Right-hand Figure (C, D, and E): Representative pseudo-coloured SIM images of a group of 70 % EtOH treated *S. aureus* biofilms grown on glass bottom petri dish, that were: (C) first stained by SYTO 9 and then (D) stained by PI, and (E) imaged again after 24 h. Scale bars in all images correspond to 1 μm. Images (C-1, D-1, and E-1) are the emission in 488 nm excitation, images (C-2, D-2, and E−2) are the emission in 561 nm excitation, and images (C-3, D-3, and E−3) are the merged images of the above two individual excitation (green for 488 nm, and magenta for 561 nm).(For interpretation of the references to colour in this figure legend, the reader is referred to the Web version of this article.)

First, control *S. aureus* biofilms were grown on glass-bottom petri dishes for 24 h, and were co-stained by both SYTO 9 and PI before observation. Fig. 3 A 1–3 show the super resolution fluorescence microscopy images in single channels and also when they are merged. These reconstructed SIM images are of much higher resolution and reveal interesting detail. Specifically, it can be seen that fluorescence intensity is unevenly distributed within the cell with the fluorescence images displaying irregular shapes and sharper edges. These details differentiate the SIM images from the largely circular cells observed in the WFM images discussed in the previous section.

SIM was then used to study 70 % EtOH treated *S. aureus* biofilms, which have been shown to generate more double-stained cells for investigation. The fluorescence excited by 488 nm laser is shown in Figs. 3 B-1, and it can be observed that most cells have uneven SYTO 9 distributions, judged by the location and the intensity of the fluorescence. A significant number of cells show strong fluorescence around the edge of the cells, with many displaying an effectively hollow fluorescence image (cell IV).

**Fig. 4:**
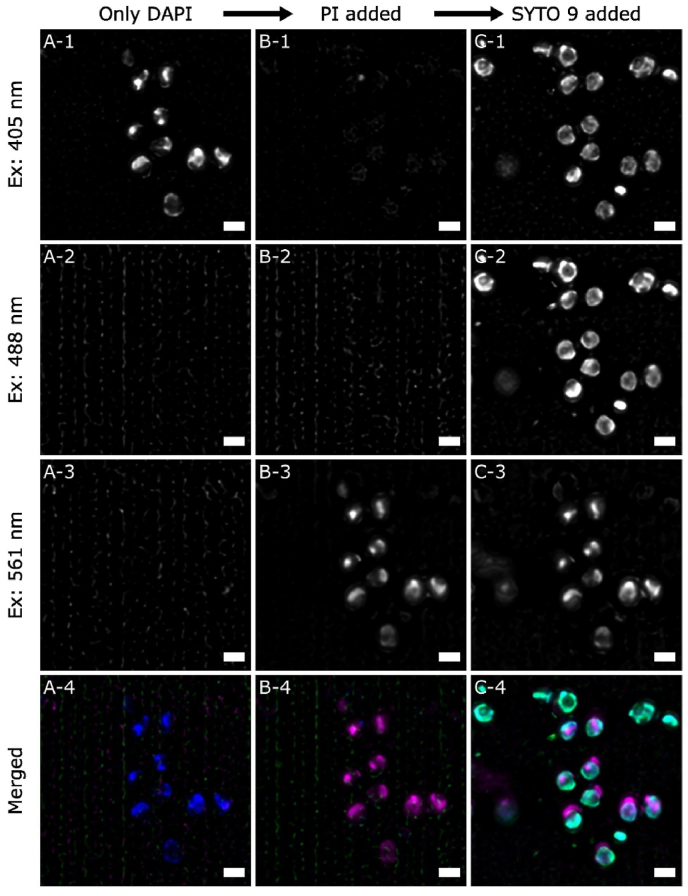
SIM data for DAPI – PI – SYTO 9 stained *S. aureus* biofilms. (A) Representative pseudo-coloured SIM images of a group of 70 % EtOH treated *S. aureus* biofilms grown on glass bottom petri dish, that were first stained by DAPI, (B) then stained by PI, and (C) finally stained by SYTO 9. Scale bars in all images correspond to 1 μm. Images (A-1, B-1, and C-1) are the emission in 405 nm excitation, images (A-2, B-2, and C-2) are the emission in 488 nm excitation, image (A-3, B-3, and C-3) are the emission in 561 nm excitation, and images (A-4, B-4, and C-4) are the merged images of the above three individual excitation (blue for 405 nm, green for 488 nm, and magenta for 561 nm).(For interpretation of the references to colour in this figure legend, the reader is referred to the Web version of this article.)

PI emission also displays irregular intensity distributions, largely located within the interior of the cell, as shown in Figs. 3 B-2. The merged data show that in some cases (cell II), the PI emission appears in the location where the SYTO 9 emission is weaker, while in others (cell III) the PI signal has overlay with the SYTO 9 signal. It has to be noted that these cells are three-dimensional objects, while the images shown here are a two-dimensional representation of the superposition of fluorescence signals. Nevertheless, these SIM images provide more detail on the locations of SYTO 9 and PI within individual cell and clearly show that SYTO 9 – PI double-staining is present in a number of cells. The quasi-wide field image (Fig. 3, Fig. 4) simulated from the SIM data shows clearly how this information is lost in a lower resolution WFM image.

#### 2.2.2 SYTO 9 – PI sequential staining

To understand the double-staining phenomenon from a different perspective, sequential staining experiments were conducted to study the PI - SYTO 9 replacement process. Here, 70 % EtOH treated *S. aureus* biofilms were first solely treated with SYTO 9 and imaged in SIM. In Figs. 3 C-1, two types of cells can be classified, based on their fluorescence distribution: some have substantially stronger intensity (cell V), and the others have weaker fluorescence and appear hollow with fluorescence emitted from the cell edge only (cell VI).

After imaging the SYTO 9 solely-stained sample, PI was added and the very same group of cells was located and imaged, as shown in Fig. 3 D. When comparing Fig. 3 D to Fig. 3 C, two significant features are noted: first, the cells that originally had stronger SYTO 9 emission (e.g. cell V, in Figs. 3 C-1) lose most of their emission when excited by 488 nm laser (Figs. 3 D-1), but gain emission when excited by 561 nm laser (Figs. 3 D-2). This suggests that PI has replaced SYTO 9 at those sites. Second, the original hollow features (e.g. cell VI) remain fluorescent when excited by 488 nm laser, showing no interaction with or replacement by PI.

To further evaluate the replacement process with time, the above samples were stored in the fridge for a further 24 h without washing (thus remained in PI-PBS medium), and the same group of cells was imaged. The sites that were stained by SYTO 9 shown in Fig. 3 E-1 remain similar to images previously shown in Figs. 3 D-1. This is also largely the same for the sites stained by PI, when comparing Fig. 3 E-2 with Figs. 3 D-2. Overall, most of the stained features remained very similar in the two sets of observations taken 24 h apart. This signifies that the stained locations do not experience any significant dye replacement over this period and also confirms the reproducibility of the SIM acquisition and reconstruction protocol.

Overall, these data suggest that PI does not replace SYTO 9 in a straightforward exchange process, as is generally assumed. The double-stained state observed here, is not the result of an intermediate state that simply requires more time for further PI diffusion or replacement. Instead, these SIM images suggest that SYTO 9 also labels some substance other than nucleic acids and that it cannot be replaced by PI in these additional sites.

#### 2.2.3 Staining with additional dyes

To further investigate these observations, additional dyes were introduced to the study. First, 4’,6-diamidino-2-phenylindole (DAPI), another commonly used dye that targets nucleic acids was used in a sequential staining experiment [26,27]. The 70 % EtOH treated *S. aureus* biofilms were first stained with DAPI only. As shown in Fig. 4 A, fluorescence is observed when excited by 405 nm laser, attributed to the DAPI-stained nucleic acids in bacterial cells.

PI was subsequently added, and the same group of cells was imaged again. Fig. 4 B shows that the original DAPI stained features were no longer excited by 405 nm laser, but by 561 nm. Importantly, the locations of fluorescence shown in Fig. 4 B-3 map well onto those previously stained by DAPI as shown in Figs. 4 A-1. This indicates that direct replacement of DAPI with PI binding to nucleic acids, occurs in a more straightforward manner than the SYTO 9 – PI replacement shown in Fig. 3.

Finally, in the last step, SYTO 9 was added to check whether further staining could be observed on the same group of cells. SYTO 9 can be excited by both 405 nm and 488 nm lasers and Fig. 4 C-1 and C-2 show that SYTO 9 does, in fact, stain the previously labelled cells, but that this occurs at the cell periphery, that are not labelled by PI (or initially by DAPI). Fig. 4 C-3 shows that PI fluorescence remains similar to that in Figs. 4 B-3, indicating that the PI stained nucleic acid sites were not affected by the addition of SYTO 9, as would be expected. In addition, we observe a number of hollow SYTO 9 single-stained cells, which did not take up either the DAPI or PI dyes. The SIM data from both types of cells clearly show that SYTO 9 does stain substances or sites that were previously not labelled by either DAPI or PI, i.e. sites not associated with nucleic acids.

A further indication on the extra binding sites of SYTO 9 is provided by FM 1–43, a lipophilic dye and believed to stain the plasma membrane [28,29]. SI Fig. 1 shows SIM images of the 70 % EtOH treated *S. aureus* biofilms that were sequentially stained by FM 1–43, PI, and SYTO 9. The strong similarity between the SYTO 9 stained sites and the FM 1–43 stained sites suggests that the extra binding sites of SYTO 9 are located closely to the plasma membrane of the cells.

## 3 Discussion

Our results show that important limitations and complexities can arise when assessing the live-dead state of bacterial cells in biofilms using imaging techniques, which can lead to difficulties in assessing survival rates and antimicrobial activity. Some of the main observations and conclusions are discussed below.

First, correlative WFM-SEM studies on early stage *S. aureus* biofilms show that SEM morphologies of *S. aureus* cells cannot be directly linked to the live-dead staining states indicated by the two dyes, SYTO 9 and PI, designed to signpost the membrane integrity and physiological states of the bacterial cells [[8], [9], [10], [11]]. As shown in Fig. 1, Fig. 2, little direct correlation can be established between the features obtained from WFM (i.e. type of dye stained, emission intensity of dyes) and the features from SEM (i.e. size, shape, and membrane integrity of the cells). For example, bacterial cells that have compromised membranes and permit PI uptake as shown in WFM, do not necessarily exhibit deformation or damage that can be clearly identified by SEM. Therefore, an intact-looking cell in SEM should not automatically be assigned to a robust physiological state. Of course, in systems where significant cell damage occurs, SEM can capture the disruption clearly [5,6]. However, where cell damage causes small and subtle changes in cell membranes and morphologies, staining of SYTO 9 and PI in WFM and SEM observation effectively provide independent information.

Second, our results show a new cause for the existence of double-stained SYTO 9 – PI bacterial cells. In an ideal co-staining experiment, bacterial cells would to be single-stained by either SYTO 9 or PI. However, in practice, double-staining of SYTO 9 and PI has been reported for different species and antimicrobial treatments via bulk sample measurements [15], flow cytometry [12] or fluorescence microscopy [8,13,14,16,17]. The double-staining phenomenon has been attributed to a number of causes by different studies. For example, it has been assigned to an intermediate bacterial state [8,15], where slow PI uptake occurs with an appreciable time-frame required for PI to completely replace the SYTO 9 at intracellular nucleic acids sites or to an extent where SYTO 9 emission can already be quenched due to FRET. The existence of double-stained cells has also been observed by CLSM [14,16], which showed that the extracellular nucleic acids (eNA), stained by PI, locate around the intact bacterial cells stained by SYTO 9. A further study [17] shows higher resolution images on individual cells, confirming a clear SYTO 9 - PI core-shell spatial distributions. The cases above impact on cell classification and either leads to overestimation of intact cells or compromised cells.

**Fig. 5:**
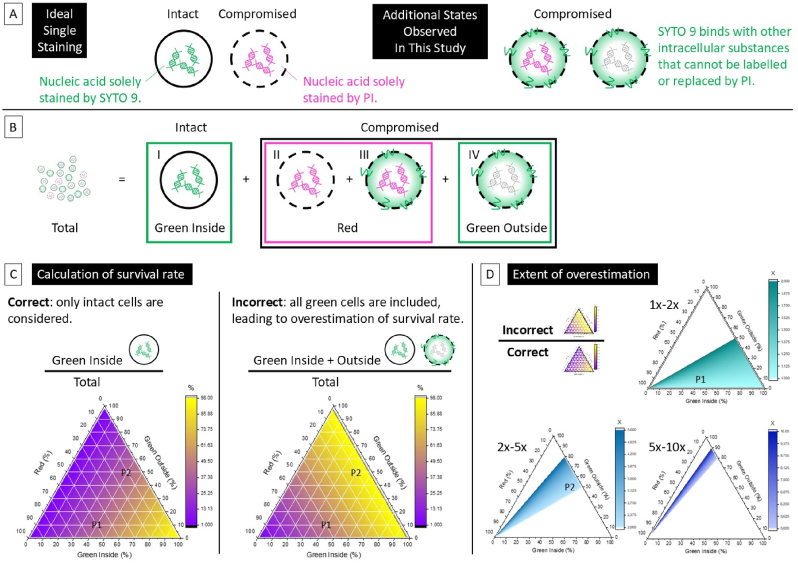
Illustration of identification of staining states and its impacts on the calculation of survival rate. (A) Schematic summary of four distinct states of SYTO 9 – PI co-stained bacterial cells observed in this study. (B) Classification of these four states in terms of the characteristic of the fluorescence observed and the indication of the physiological state of the cells. (C) Two ternary plots representing the correct and possible incorrect calculation of the survival rate. The white grids provide guide to establish the actual combinations of the cells present and the colour at each position indicates the perceived survival rate as indicated by the bar chart. (D) The extent of possible overestimation presented in a set of ternary plots for ranges of overestimation: 1x-2x; 2x-5x; 5x-10x.(For interpretation of the references to colour in this figure legend, the reader is referred to the Web version of this article.)

In this study, the application of super resolution SIM revealed a new cause of SYTO 9 – PI double-staining that is different from previous reports. The main aspects are summarised in Fig. 5 A. Spatially resolved distributions of SYTO 9 and PI of double-stained *S. aureus* cells show the PI signal is encircled by the SYTO 9 signal, reminiscent of the core-shell distribution reported for eNA [14,16,17], but with the opposite configuration. This double-staining is not due to a slow PI - SYTO 9 replacement process, since prolonged staining of PI does not substantially change the SYTO 9 stained sites, thus excluding the intermediate state explanation [8,15]. Co-staining results using a different nucleic acid dye DAPI showcase a clear example of direct replacement by PI. Importantly, subsequent addition of SYTO 9 to a DAPI-PI co-stained biofilm demonstrates that SYTO 9 has the ability to stain other substances, apart from nucleic acids, which are located at the cell perimeter and are not binding targets for either DAPI or PI. Experiments using FM 1–43, a lipophilic dye, show very similar distributions to those exhibited by the additional SYTO 9 staining. We, therefore, attribute this additional SYTO 9 staining near or at the cell membrane as the main cause of the SYTO 9 – PI double-stained *S. aureus* cells observed in this study.

The potential of SYTO 9 in binding to substances at the cell perimeter also produces cells that are single-stained by SYTO 9 around the membrane, exhibiting a hollow-shaped fluorescence emission. Such cells show no staining by either DAPI or PI, signifying that the intracellular nucleic acids may have already leaked or been degraded after the 70 % EtOH treatment. In either case, these cells should be classified as compromised cells, similar to “ghost” cells reported previously using scanning transmission electron microscopy [30]. However, they would show as fluorescent green in a lower resolution WFM image, leading to a misclassification.

Our high resolution SIM experiments effectively show that four distinct types of staining can be found in cells that were co-stained by SYTO 9 and PI, as depicted in Fig. 5 A&B: (I) single-stained cells that appear fluorescent green within the intracellular space; (II) single-stained cells that appear fluorescent red within the intracellular space; (III) double-stained cells that possess a mixture of red and green fluorescence; (IV) single-stained cells that only appear fluorescent green at the cell edge. Here we simply group states (II) and (III) together as “red”, since both represent cells that are stained by PI and whose red fluorescence should be detected by conventional WFM and classified as compromised. On the other hand, it is important to distinguish cell type (I) from cell type (IV), because, although they both emit fluorescent green, only type (I) should be regarded as intact, while type (IV) is compromised. Whereas this differentiation is possible in super resolution SIM images, these states are not easily distinguishable in a conventional WFM experiment. For example, in the quasi-WFM images shown in Fig. 3, Fig. 4, it is difficult to distinguish cell I (type I) from cell IV (type IV). Failing to differentiate and count these two states separately results in overestimation of intact cells, that is the overestimation of the survival rate.

To illustrate how these different staining states can alter the evaluation of survival rate, we designed some ternary plots for discussion. As shown in Fig. 5 C, there are three components/axis in these ternary plots, representing the percentage of cells with green fluorescence inside (type I), red fluorescence (types II and III), and green fluorescence on the perimeter (type IV). Each position within the ternary plot represents a unique combination of these three states that exists for the system in the actual scenario. However, the experimental readout of survival rate at each position depends critically on whether the states are correctly distinguished. For example, a correct evaluation of the survival rate should distinguish and consider the population of cells that are intact and have green fluorescence inside (type I) only. This scenario is depicted in the ‘Correct’ ternary plot, Fig. 5 C, where the survival rates for each combination is calculated correctly and then colour coded from purple (0 % survival) to yellow (100 % survival). For illustrative purpose, two different positions P1 and P2 on the plots are marked. One can clearly see that although they are composed of different ratios of the three states (40 %:50 %:10 % at P1 vs 40 %:10 %:50 % at P2), they constitute the same survival rate of 40 % since the percentage of intact type I cells in both populations is the same and, therefore, share the same colour coding at both positions.

However, if one is not able to distinguish between type I and type IV SYTO 9 stained cells (a likely scenario in WFM imaging), then all the green cells would be counted as intact with consequent overestimation of the survival rate. This is depicted in the ‘Incorrect’ ternary plot in Fig. 5 C, where cells type I and type IV are both assigned to the live state. This leads to incorrect survival rates for each combination within the ternary plot, as shown by the colour-coding assigned to each position. Comparing the same marked positions, P1 and P2, used in the ‘Correct’ plot, it can be seen that they are now coded with different colours in the ‘Incorrect’ plot and give an overestimated survival rate of 50 % at P1 and 90 % at P2, as opposed to the correct survival rate of 40 %. To further illustrate the extent of overestimation, the ratios of the incorrectly calculated survival rate versus the correct one are shown in Fig. 5 D. Three different regions of overestimation on the ternary plot are shown separately for clearer display of the areas where overestimation ranges from 1x-2x (e.g. 1.25x for P1), from 2x-5x (e.g. 2.25x for P2), and finally 5x-10x. Therefore, depending on the actual combinations of cells in a system, very different extents of overestimation can occur.

The ternary plots in Fig. 5 show that ability of SYTO 9 to interact with substances other than nucleic acids significantly compromises its ability to act as a true counterstain to PI, which only replaces SYTO 9 on nucleic acids. Therefore, alternative dyes that only stain nucleic acids may offer better options as a counter-stain to PI and to indicate the membrane integrity. From the results shown in Fig. 4, DAPI appears to be a good candidate for our system as it only stains the sites that PI can bind with, and is directly replaced by PI when they are sequentially stained. Further investigation on the concentrations of dyes can also be meaningful, which could indicate whether it is possible to find a sweet spot that allows true counterstain to occur.

We note that using nucleic acid co-staining methods to identify the states of cells still has its natural blind spot, because cells can become compromised in various ways, including situations where they lose their nucleic acids entirely and, therefore, would not be labelled. From this perspective, the additional staining ability of SYTO 9 found in this study on *S. aureus* provides an advantage, since it reveals the existence of such compromised cells. Our early work on *E. coli* suggests that such double-staining may also be important in Gram-negative bacteria and this is currently being pursued. In conclusion, fluorescence microscopy and the SYTO 9 – PI co-staining method remains a widely used option to investigate membrane integrity and the survival of bacterial cells, and does indeed provide a valuable overview of whether significant antimicrobial activity is present. However, for careful analysis of survival rates, there is a clear merit in using objectives with higher magnification and higher numeric aperture, or even better, a super resolution microscopy technique to better map the distribution of dyes within the cell in order for cells to be correctly classified.

## 4 Methods

### 4.1 Preparation of substrates

For the WFM-SEM correlative study, single side polished silicon wafers (p-type, (100)-orientation) and circular coverglass (agarscientific, AGL46R13-1), were used as substrates to grow biofilms. Before each usage, they were cleaned with ethanol in an ultrasonic bath for 15 min and aseptically dried near a Bunsen burner. For the SIM studies, ibidi μ-Dishes (35 mm, high) with Grid-50 Glass Bottom were used as substrates for biofilm growth.

### 4.2 Growth of biofilms

*Staphylococcus aureus* ATCC 6538P (*S. aureus*) was transferred from the −80 °C frozen stock to nutrient broth (NB, Sigma) agar plate and incubated overnight at 37 °C. A colony was taken from the agar plate with a sterile metallic loop and transferred to 5 mL Luria-Bertani broth (LB, BD) medium in a 50 mL Falcon tube. It was then incubated aerobically overnight at 37 °C with a speed of 180 rpm. The next day, the overnight culture was diluted 1:1000 in fresh LB medium. For the WFM-SEM correlative study, the corresponding sterile substrates were transferred into a 24-well plate, to which 1 mL of the diluted bacterial suspension per well was added. For the SIM studies, 300 μL of the diluted bacterial suspension was pipetted onto the centre of the μ-Dish. The 24-well plate and the μ-Dish were then sealed with parafilm and incubated statically at 37 °C for 24 h.

### 4.3 Widefield Fluorescence Microscope (WFM) imaging

The FilmTracer LIVE/DEAD Biofilm Viability Kit (ThermoFisher) was used. After defrosting at room temperature, 1.5 μL SYTO 9 (3.34 mM) and 1.5 μL propidium iodine (PI, 20 mM) were mixed with 1 mL phosphate buffer saline (PBS, Sigma), producing 1 portion of dye mixture.

Following the 24 h incubation for biofilms growth in the 24-well plate, each sample was washed within its well with PBS for three times. For, the non-treated samples, the last washed PBS was withdrawn and 1 mL of the dye mixture was added. The samples that were designed to be treated with 70 % EtOH were immersed for 10 s in fresh wells with 1 mL 70 % EtOH, and then transferred to fresh wells with 1 mL of the dye mixture. After 15 min incubation, the samples were again washed with PBS for three more times, and then transferred to a PBS filled petri dish for imaging.

A Zeiss Axio Imager 2 upright fluorescence microscope was used for imaging. A 40x water dipping objective with NA 1.0 was used. Excitation was provided by solid state LED lamps, with a 469/38 nm (wavelength/bandwidth) LED was used to excite SYTO 9, while a 555/30 nm LED was used to excite PI. A multi-pass filter set (Zeiss 90 HE) was applied to filter the emission light, allowing 425/30 nm, 514/30 nm, 592/25 nm, and 709/100 nm to pass through, at all the times. A 1.4 megapixels CCD camera was used to record the images. Images shown in the figures were adjusted with the Zen (Zeiss) built-in curve tool, no gamma adjustment was applied. Pseudo colours were assigned to the merged images: green for the emissions under excitation of 469 nm LED, and magenta for emissions under excitation of 555 nm LED.

### 4.4 Scanning Electron Microscope (SEM) imaging

Following the WFM imaging, the samples were then transferred back to their own wells. The PBS was then withdrawn and 1 mL fixative (4 % paraformaldehyde, 2.5 % glutaraldehyde in 0.1 M phosphate buffer) was added. The well plate was then sealed with parafilm and stored at 4 °C overnight. The next day, the samples were washed three times with Milli-Q water, and further fixed with 2 % osmium tetroxide and accelerated in a microwave (Pelco Biowave Pro). After that, the samples were washed with Milli-Q water for another three times, and then progressively dehydrated by replacing the water with a series of ethanol in increasing concentrations (30 %, 60 %, 90 %, 100 %, 100 %, 100 %). CO_2_ critical point dryer (Quorum K850) was then used to remove the ethanol. Last, the samples were sputtered coated (Quorum Q150T) with 10 nm Au/Pd for conductivity. A scanning electron microscope (JEOL JSM-7001F) was then employed at 5 kV to collect images with through-the-lens (TTL) Everhart-Thornley detector.

### 4.5 Structured Illumination Microscope (SIM) imaging

In addition to the SYTO 9 and PI dye mixture mentioned in Section 4.4, 1.5 μL SYTO 9 (3.34 mM) was mixed with 1 mL PBS, producing 1 portion of SYTO 9 dye. A 400-fold PBS diluted DAPI from ViaGram™ Red + Bacterial Gram Stain and Viability Kit (Thermofisher) was also prepared, producing 1 portion of DAPI dye.

Following the 24 h incubation for biofilms growth in the μ-Dish, the dish was washed three times with 500 μL PBS. For the control, another three washes with PBS were undertaken. For the samples that were designed to be treated with 70 % EtOH, 500 μL 70 % EtOH was applied for 30 s, and then washed again with 500 μL PBS for three times. The samples were then ready using the following staining protocols.

For the direct SYTO 9 – PI co-staining, 500 μL of the dye mixture was added. After 15 min incubation, the samples were again washed with PBS for three more times before imaging.

For the SYTO 9 – PI sequential staining, 500 μL SYTO 9 dye was added. After 15 min incubation, the samples were washed again with PBS for three more times before imaging. After the first round of imaging, 0.5 μL PI (20 mM) was added directly to the μ-Dish and imaged instantly. After the second round of imaging, the μ-Dish was stored in the fridge for another 24 h, and then imaged for the third time.

For the DAPI – PI – SYTO 9 sequential staining, 200 μL DAPI dye was added. After 30 min incubation, the samples were washed again with PBS for three times before imaging. After the first round imaging, 0.5 μL PI (20 mM) was added directly to the μ-Dish and imaged instantly. After the second round imaging, 0.5 μL SYTO 9 (3.34 mM) was added directly to the μ-Dish and imaged instantly.

A Zeiss Elyra 7 inverted structured illumination microscope was used for imaging. A 63x oil immersion objective with NA 1.4 was used. Excitation was provided by lasers, with 405 nm used to excite DAPI (and SYTO 9), 488 nm used to excite SYTO 9, while 561 nm used to excite PI. The filter set SBS LP 560 was applied to filter the emission light, allowing 570–620 nm and from 655 nm to pass through to the camera 1 (used in 561 nm excitation), while 420–480 nm, 495–550 nm are reflected to the camera 2 (used in 405 nm and 488 nm excitation). Each acquisition contains 15 phases of raw image. These were then reconstructed with SIM [2] algorithms, with the following parameters: iterations 20, regularization weight 0.04, processing sampling 4x, output sampling 4x, filter Median, intensity scaled to raw image. Images were then adjusted with the Zen (Zeiss) built-in curve tool, no gamma adjustment was applied. Pseudo colours were assigned to the merged images: blue for emissions under excitation of 405 nm laser, green for the emissions under excitation of 488 nm laser, and magenta for emissions under excitation of 561 nm laser.

## CRediT authorship contribution statement

**Jiaqi Luo:** Writing – original draft, Visualization, Investigation, Conceptualization. **Rasmita Raval:** Writing – review & editing, Supervision, Resources, Funding acquisition, Conceptualization.

## Competing interests statement

The authors declare that they have no conflict of interest.

## Data Availability

The data that support the findings of this study are available from the corresponding author on request.
