# Supplementary material for: Correlative Imaging and super resolution microscopy studies reveal complexities in determining live-dead state of bacteria
Source: Biofilm. 2025 Jul 3;10:100302. doi: 10.1016/j.bioflm.2025.100302 (PMC12284284; doi:10.1016/j.bioflm.2025.100302)
Supplement: Multimedia component 1 [file mmc1.pdf]

# Supplementary Information

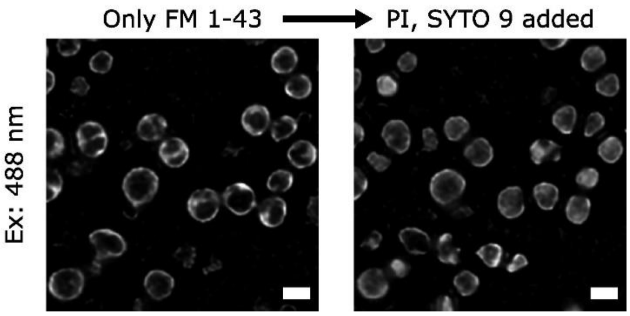


SI Figure 1

Representative pseudo-coloured SIM images of 70% EtOH treated *S. aureus* biofilms grown on glass bottom petri dish, that were first stained by FM 1-43 and then stained by PI and SYTO 9. Both images display the emission in 488 nm excitation, with scale bars that correspond to 1 um.
